# Analysis of Facial Features according to Sasang Types between Native Japanese and Native Korean Populations

**DOI:** 10.1155/2018/6950216

**Published:** 2018-08-01

**Authors:** Lin Ang, Jong Yeol Kim, Jeongyun Lee

**Affiliations:** ^1^Korea Institute of Oriental Medicine (KIOM), 1672 Yuseong-daero, Yuseong-gu, 34054 Daejeon, Republic of Korea; ^2^Korean Medicine Life Science, University of Science and Technology, 217 Gajeong-ro, Yuseong-gu, 34113 Daejeon, Republic of Korea; ^3^Division of Clinical Medicine, School of Korean Medicine, Pusan National University, 49 Busandaehak-ro, Mulgeum-eup, Yangsan-si, 50612 Gyeongsangnam-do, Republic of Korea

## Abstract

**Background:**

Facial diagnosis is a common practice and essential diagnostic method used in the Sasang Constitution Medicine (SCM). SCM is a kind of personalized medicine in Traditional Korean Medicine which categorizes people into four types, namely, Tae-Yang (TY) type, Tae-Eum type (TE), So-Yang (SY) type, and So-Eum (SE) type. This study was conducted to compare and analyze the differences in the facial feature across Sasang types among native Japanese and native Koreans.

**Methods:**

A total of 843 subjects were recruited for this study, 127 native Japanese and 716 native Koreans, respectively. Facial feature points and the measurements of facial features were assigned and calculated automatically using a facial analysis program. Data of each Sasang type for both genders were also extracted and analyzed. Analysis of covariance was then used to examine the differences in facial feature variables among native Japanese and native Koreans and Sasang types.

**Results:**

Significant differences were seen in the facial feature variables related to lower face area and eye shape. In males, TE types had wider mid-face and lower face as compared to other constitutions. Male TE types were also seen to have narrower eyes whereas male SY types had rounder eyes. In females, TE types had wider lower face width and area compared to SY types and SE types. Female SY types also had rounder eyes.

**Conclusions:**

This study presented distinctive feature in the lower face area and eye shape among the Sasang types in both native Japanese and native Koreans. This proposed that facial feature variables can also be used as an objective tool in distinguishing the Sasang types in native Japanese. Further studies are needed in the future to generalize these results.

## 1. Background

Sasang Constitutional Medicine (SCM) is a kind of typological personalized medicine in Traditional Korean Medicine which categorized people into four types, Tae-Yang (TY) type, Tae-Eum (TE) type, So-Yang (SY) type, and So-Eum (SE) type. This theory was established and written in the classic Longevity and Life Preservation in Eastern Medicine by Lee Jema around the year 1894 [1]. According to this classic, the determination of Sasang type comprised several diagnosis elements such as facial appearance, type of body shape, biopsychological traits, pathophysiological symptom diagnosis, and type-specific clinical response [2–4].

In the midst of those diagnosis elements, facial appearance can be regarded as one of the most important aspects [5]. Although SCM classics had descriptions of the facial characteristics of the four Sasang types, those statements are relatively subjective and less quantitative [1, 6–13]. Therefore, many efforts had been made to standardize and objectify the diagnosis of Sasang types using facial features [14–22].

In addition, SCM specialists had strongly accounted for facial features as one of the most reliable elements in distinguishing Sasang types [23]. Many studies had been conducted and facial features were shown to be highly employed in the determination of Sasang types [6, 16, 24–26]. Another study had also mentioned that face shape was found to be more utilized among all the facial elements. Recently, a standardization approach on the facial diagnosis of Sasang types according to quantitative analysis of facial features was established and the representative facial images of each Sasang type among native Koreans were made public in the year 2012 [5, 27].

The purpose of this study was to expand the usage of facial features in the determination of Sasang type towards other ethnicities. We investigated and compared the facial features among native Japanese and native Koreans across Sasang types by collecting their facial photographs and analyzing them quantitatively.

## 2. Methods

### 2.1. Subjects

#### 2.1.1. Native Japanese (Sample A)

This study was conducted at Tohoku University, Sendai, Japan, from 2010 to 2011 after receiving approval from the Institutional Review Board of Tohoku University where a total of 127 native Japanese were recruited. All the subjects who were eligible met the following inclusion criteria: (1) age ranging from 18 to 40 years and (2) being healthy and not suffering from chronic diseases. Subjects who have undergone plastic surgery or facial reconstruction surgery due to trauma were excluded. Male subjects with excessive facial hair were also excluded. A written consent form was signed by all the subjects before participating in this study.

#### 2.1.2. Native Korean Subjects (Sample B)

Data collected by Korea Constitutional Multicenter Bank (KCMB) between 2007 and 2010 were used in this study. Data of 716 native Koreans whom their age ranged from 18 to 40 years were extracted and analyzed. This procedure was done with the approval of the Korean Institute of Oriental Medicine Institutional Review Board (I-0910/02-001).

### 2.2. Classification of Sasang Types

The determination of Sasang types for both native Japanese and native Korean was performed by trained experts using Sasang Constitutional Analysis Tool (SCAT) under standard operating procedures, where the SCAT system analyzed the combination of information on facial images, body shape, voice, and questionnaires [20, 22]. In terms of questionnaires, the Korean language questionnaires developed for the use of SCAT were translated into Japanese language and the reliability assessment of the questionnaire was performed [28].

In order to further confirm the Sasang constitutions of the subjects, the Sasang constitutions of native Japanese subjects were confirmed by a certified Sasang medicine specialist (JY Kim) who has more than 8 years of clinical experience accompanied by a professional translator whereas Sasang constitutions of native Korean subjects were then further determined based on the response of subjects towards Sasang type-specific herbal medicine. Sasang constitution of the native Korean subjects was confirmed by a certified Sasang medicine specialist when they showed improvements in ordinary symptoms and did not suffer from adverse effects after taking the prescription for 50 or more days.

### 2.3. Facial Photography

Frontal full face and profile pictures are essential and should be taken with a neutral facial expression for all subjects. Pictures were taken at a fixed subject-camera distance of 1.6 m using a Nikon D700/D5100 digital camera with 85 mm lens under bilateral illumination. The camera was maintained at the same height as each subject. Images were taken at a resolution of 3184 × 2120 pixels in JPEG format using 24-bit RGB encoding. As for the subjects, ears and hairline of each subjects have to be revealed using a hair tie or hair band during the photography shoot. A ruler used for converting pixels into millimeters was placed approximately 1 cm below the chin. The first shot is the full face frontal view. Subjects were instructed to look at the lens of the camera with their heads positioned in a way that the central point of the two pupils and the upper auricular points were horizontal. The next shot is the profile view where the subject's face is turned approximately 90 degrees from the front. Only one side of their face and not the eye on the far side should be seen. The central point of the pupil from the side and point of upper auricular should also be on the same horizontal line.

### 2.4. Measurement of Facial Feature

**Figure 1:**
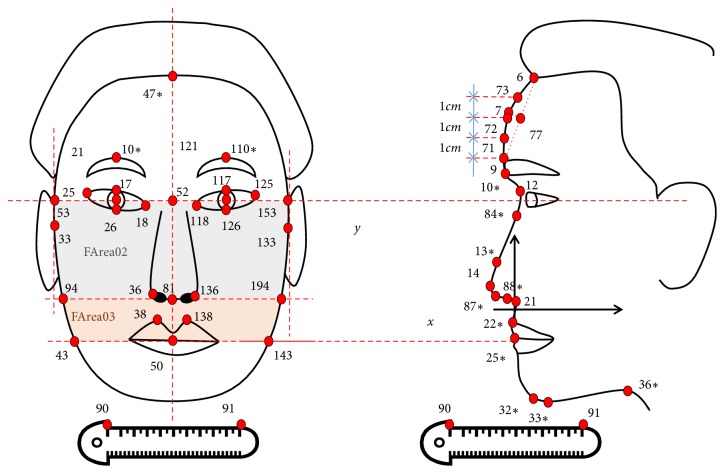
Automatically allocated facial feature points using facial analysis program [29].

The facial feature points were automatically allocated by uploading the facial images into the Sasang Constitutional Analysis Tool (SCAT) as shown in Figure 1. Facial feature variables were also automatically calculated using length, length to length ratio, angle, and area between facial feature points (Table 1).

### 2.5. Statistical Analysis

Data analysis of facial features was conducted independently for each sample according to gender. The differences in the general characteristics (age, height, weight, and BMI) of both samples were tested using Student's* t*-test. The differences in general characteristics (age, height, weight, and BMI) of Sasang types for native Koreans were tested using one-way ANOVA with Bonferroni or Dunnett's T3 as post hoc analysis, depending on the result of Levene's test. For native Japanese, general characteristics (age, height, weight, and BMI) of Sasang types were tested with Kruskal-Wallis test with Mann–Whitney as post hoc analysis. Statistical results were presented as mean (standard deviation).

The facial features of the samples were analyzed according to the Sasang types using one-way ANCOVA, with age as covariates and sample and Sasang types as factors. Post hoc analysis was performed with a Bonferroni adjustment. Statistical results were presented as adjusted mean (standard error).

Statistical analyses were performed using IBM SPSS Statistics 23.0 for Windows (IBM, Armonk, New York) at the significant level of 0.05 as the *p* value.

## 3. Results

### 3.1. General Characteristics of the Subjects

The general characteristics of the subjects by samples were shown in Table 2. The mean age of native Japanese (Sample A) was 23.8 ± 4.4 years (ranging from 20 to 40 years old) for males and 24.2 ± 5.4 years (ranging from 19 to 40 years old) for females. The mean age of native Koreans (Sample B) was 31.2 ± 6.1 years (ranging from 18 to 40 years old) for males and 31.4 ± 5.9 years (ranging from 18 to 40 years old) for females. In both males and females, age, height, body weight, and BMI were significantly higher in Sample B than in Sample A.

The general characteristics of the samples' Sasang types were shown in Table 3. There were no significant differences in age and height among the Sasang types for both genders in Sample B and females only in Sample A. For males in Sample A, there was no significant difference in age but there were significant differences in height. However, there were significant differences in weight and BMI among the Sasang types for males and females in both samples. Weight and BMI for both samples were the highest in TE types and lowest in SE types according to post hoc analysis.

### 3.2. Differences in Facial Features among the Samples and Sasang Types

After adjustment for age, there were several statistically significant differences in the facial features variables among samples and Sasang constitution in both genders.

#### 3.2.1. Difference in Facial Features in Males

In males, lower face angle and area variables such as FA_18_17_43, FA_118_117_143, and FArea03_aD showed significant differences between the samples and also among the Sasang constitutions. Lower face area was larger in Sample B and also in the TE types as compared to SE types and SY types. Mid-face area variable, FArea02_aD, was also seen significantly different where the mid-face of Sample B is larger than Sample A. The value of this variable was also highest in TE types compared to SE types and SY types (Table 4).

There was also a significant difference in the eye horizontal distance variable, FDH_25_125, and also in the variables related to eye slanting angle, FA_18_17_25 and FA_118_117_125. This showed that the outer eye horizontal distance and eye slanting angle were larger in Sample B and also in TE types. Moreover, inner eye slanting angle, FAis_18_17 and FAi_118_117, was larger in Sample A and also in SY types as compared to other Sasang types (Table 4).

Forehead related variable, PD_7_77, was also significantly different across samples and Sasang types. The forehead of Sample A and SY types was more protruding than Sample B and other Sasang types. Furthermore, there is also a significant difference in the nose related variable such as PDH_12_14 where Sample A had shorter nose than Sample B. For this variable, the noses of SY types were shorter than SE types (Table 4).

#### 3.2.2. Difference in Facial Features in Females

In females, the facial variable related to the distance between the root of nose and lips, FDV_52_50, and philtrum related variable, FDV_81_50, showed significant differences among the samples. The values of these variables were larger in Sample A than Sample B which suggested that Sample A had longer nose and philtrum (Table 5).

Lower face angle and area variables, FA_18_17_43 and FArea03_aD, also showed significant differences among the samples and Sasang constitutions. This indicates that lower face width and area was overall wider in Sample A and in the TE types compared to the SY and SE types. There was also significant difference seen in variable FD_17_26, which is the distance between upper and lower eyelid in vertical alignment. Furthermore, variable related to the angle of eye slanting, FAis_18_17, also showed significant differences where Sample B and SE types had a wider angle than Sample A and other Sasang types (Table 5).

## 4. Discussion

As the diagnosis of facial features is one of the fundamental aspects of SCM, understanding the differences in facial features among Sasang types is essential. Currently, the standard approach to Sasang types facial diagnosis among native Koreans has already been established using quantitative analysis. However, the use of Sasang types facial diagnosis in other populations is still limited. This study was performed to extend the use of Sasang facial diagnosis in native Japanese.

In this study, we analyzed the differences in facial feature variables among the samples and Sasang types. In terms of demographic characteristics, we found that the age, height, weight, and BMI were significantly higher in Sample B than in Sample A for both genders (Table 2). The average height and weight of our samples correspond with the average height and weight of both nationalities in general, which could suggest that the results in our study could speak for native Japanese and native Koreans in general [30–34]. Besides, TE types also had the highest weight and BMI followed by SE types as the lowest in both samples (Table 3). This shows that the results of Sample A are consistent with the previous study of Sample B where TE type was reported to have highest BMI score and it was lowest in SE type [4, 17, 35].

After adjustment for age, facial variables such as FA_18_17_43, FArea03_aD, and FAis_18_17 were the common variables significantly different in both males and females. Therefore, lower face variables and eye-related variables were considered the main indicators that distinguish the Sasang types in both genders among both samples. The value of lower face angle and area variables, FA_18_17_43 and FArea03_aD, was higher in male and lower in the female of Sample B indicating that lower face of Sample B was larger than Sample A in male and vice versa in the female. However, the values of these variables were higher in TE type of both males and females (Tables 4 and 5). These results are similar to those previous studies that analyzed facial feature according to Sasang types among native Koreans. It was mentioned that the width over length was larger in TE type with the width of jaw serving as standard and generally TE type had a wider jaw than other Sasang types [16, 17, 25].

On the other hand, the value of the variable related to the area of mid-face, FArea02aD, was also higher in the male of Sample B and TE types (Table 4). This indicates that male TE types have larger mid-face. Looking at the results of both mid-face and lower face area variables for males, we can suggest that males of TE types have wider face area compared to other Sasang types. These results could be considered consistent with the previous studies where the face of TE types was mentioned to be larger than other types [16].

Furthermore, the value of eye slanting angle variables, FA_18_17_25 and FA_118_117_125, was higher in the males of Sample B compared to the males of Sample A and vice versa for the inner eye slanting angle variables, FAis_18_17 and FAi_118_117. This indicates that Sample A males have rounder eyes whereas Sample B males have narrower eyes. However, in females, the value of inner eye slanting angle variable FAis_18_17 and the value of variable FD_17_26, which is the vertical distance of upper and lower eyelid, were higher in Sample B (Tables 4 and 5). This shows that Sample A females have narrower eyes and Sample B females have rounder eyes. Our findings showed that the eye shape of both genders in Sample A and Sample B is opposite to each other.

In terms of Sasang types, the value of eye slanting angle variables, FA_18_17_25 and FA_118_117_125, is the highest in TE types compared to other types in males. This shows that TE types of males have narrower eyes. In addition, males of SY types and females of SE types have the highest value in the inner eye slanting angle variable, FAis_18_17, indicating that SY types males and SE types females have rounder eyes (Tables 4 and 5). A previous study had stated that female TE types had narrower eyes and female SE type had rounder eyelids [25]. Another study also suggested that the eye of SE type is round in shape and the eye of SY type was relatively rounder than TE types [16]. Our results showed similar consistency with previous studies. Moreover, the value of forehead related variable, PD_7_77, is highest in SY types according to our post hoc analysis. This finding also matched the descriptions of previous studies which mentioned that the SY types had the most protruding forehead and bulging head [23, 25].

We also found that there are a few significant results when we look only at the differences among the Sasang types without regard to the differences in samples. In males, there were significant differences in the variable PDV_14_21 among the Sasang types. This showed that TE types have longer vertical distance between the apex of nose and subnasale. This results indicated that, regardless of sample types, the nose of TE types males was more turn-up compared to other types.

In addition, the consistency between our study and previous studies implies that the algorithm used in Sasang Constitutional Analysis Tool for native Koreans can also be utilized in the analysis of Sasang types of native Japanese provided that the standardized values compensating for the difference of sample size are assigned to the algorithm [22]. Hence, further studies are needed to validate our findings.

There are several limitations in this study. Firstly, the determination of the subjects' Sasang types is inconsistent between the samples. Sasang types of the subjects in Sample B were decided based on their response towards Sasang type-specific herbal medicine while Sasang types of the subjects in Sample A were determined by only one certified clinical specialist and they were not prescribed Sasang type-specific herbal medicine. Therefore, there might be a question on the generality of data. Secondly, subjects in Sample B were chosen from data bank based on the predefined inclusion criteria whereas subjects in Sample A were recruited from one university, resulting in unequal sample size which may affect the ability to generalize our findings. Although we did necessary statistical adjustment for age, conclusion involving these data should be drawn with caution.

Hence, future studies should include a larger sample size from various places of Japan and Sasang types of subjects should be determined by at least two or more certified clinical specialists with their interrater reliability tested. The improvement in the reliability of data collection will greatly improve the accuracy of our research. Such follow-up studies will greatly generalize our findings and may be able to yield new discoveries.

## 5. Conclusion

This study is the first study which attempts to analyze the facial features of native Japanese individuals according to Sasang types. Although there were differences in facial features among Sample A and Sample B, the facial features of both samples across Sasang types showed a similar tendency. If the distinctive variables are applied after compensating for the differences between samples, the Sasang Constitutional Analysis Tool may be valid and usable in distinguishing the Sasang types of native Japanese.

## Figures and Tables

**Table 1:** Description of facial feature variables.

Variables	Description
FD_*n1_n2 *[or PD_*n,_n2*]	The length between two points in the frontal (side) picture
FDH_*n,_n*2 [or PDH_*n1_n*2]	The horizontal length between two points in the frontal (side) picture
FDV_*n1_n*2 [or PDV_*n1_n*2]	The vertical length between two points in the frontal (side) picture
FDL_*n1_n*2*_n*3 [or PDL_*n1_n*2*_n*3]	The length between the point *n1 *and segments *n2*, *n3*
FHD_*n1_n*2*_n*3*_n4 *[or PHD_*n1_n*2*_n*3*_n4*]	FDH_*n1*_*n2 */ FD_*n3*_*n4 *[or PDH_*n1*_*n2 */ PD_*n3*_*n4*]
FDH_*n1_n*2*_n*3*_n4 *[or PDH_*n1_n*2*_n*3*_n4*]	FD_*n1*_*n2 */ FDH_*n3*_*n4 *[or PD_*n1*_*n2 */ PDH_*n3*_*n4*]
FDD_*n1_n*2*_n*3*_n4 *[or PDD_*n1_n*2*_n*3*_n4*]	FD_*n1*_*n2 */ FD_*n3*_*n4 *[or PD_*n1*_*n2 */ PD_*n3*_*n4*]
FVD_*n1_n*2*_n*3*_n4 *[or PVD_*n1_n*2*_n*3*_n4*]	FDV_*n1*_*n2 */ FD_*n3*_*n4 *[or PDV_*n1*_*n2 */ PD_*n3*_*n4*]
FVV_*n1_n*2*_n*3*_n4 *[or PVV_*n1_n*2*_n*3*_n4*]	FDV_*n1*_*n2 */ FDV_*n3*_*n4 *[or PDV_*n1*_*n2 */ PDV_*n3*_*n4*]
FVH_*n1_n*2*_n*3*_n4 *[or PVH_*n1_n*2*_n*3*_n4*]	FDV_*n1*_*n2 */ FDH_*n3*_*n4 *[or PDV_*n1*_*n2 */ PDH_*n3*_*n4*]
FA_*n1_n2 *[or PA_*n1_n2*]	The angle that the straight-line vector (n1, n2) makes with the horizontal line in the frontal (side) image
FAs_*n1_n2* [or PA_*n1_n2*]	180—The angle that the straight-line vector (n1, n2) makes with the horizontal line in the frontal (side) image
FAi_*n1_n2* [or PAi_*n1_n2*]	The angle that the straight-line vector (n1, n2) makes with the horizontal line in the frontal (side) image *∗* (-1)
FAis_ *n1_n2* [or PAis_*n1_n2*]	180—The angle that the straight-line vector (n1, n2) makes with the horizontal line in the frontal (side) image
FA_*n1_n2_n3* [or PA_*n1_n2_n3* ]	The angle formed by the three points n1, n2, and n3 in the frontal (side) image
FArea02	The area of the face defined using points 53, 94, 194, and 153
FArea03	The area of the face defined using points 94, 43, 143, and 194

**Table 2:** General characteristics of the subjects by samples.

	Males			Females		
	Sample A	Sample B	*P* value	Sample A	Sample B	*P* value
	(n = 61)	(n = 252)		(n = 66)	(n = 464)	
Age (yrs)	23.83 (4.39)	31.18 (6.08)	<0.001	24.19 (5.36)	31.43 (5.93)	<0.001
Height (cm)	170.80 (6.06)	173.50 (5.67)	0.001	158.67 (5.28)	160.92 (5.18)	0.001
Weight (kg)	64.93 (9.13)	72.54 (11.23)	<0.001	52.27 (6.37)	55.81 (8.89)	<0.001
BMI (kg/m^2^)	22.20 (2.48)	24.36 (3.14)	<0.001	20.75 (2.22)	21.55 (3.24)	0.012

Sample A: native Japanese, Sample B: native Koreans, and BMI: Body Mass Index.

Data were presented as the mean (standard deviation). *P* values were calculated using *t*-test.

**Table:** (a) Sample A

Sample A (n = 127)	Male (n = 61)				Female (n = 66)			
	TE (n = 17)	SE (n = 35)	SY (n = 9)	*P* value	TE (n = 9)	SE (n = 19)	SY (n = 38)	*P* value
AGE (yrs)	22.34 (1.72)	24.03 (4.66)	25.84 (6.08)	0.320	22.82 (4.29)	22.71 (4.78)	25.26 (5.70)	0.127
Height (cm)	174.07 (5.47)^a^	169.42 (6.42)^b^	169.99 (3.01)	0.022	158.27 (7.23)	158.82 (4.58)	158.70 (5.23)	0.855
Weight (kg)	72.30 (9.27)^a^	60.60 (6.72)^b^	67.82 (7.34)^a^	<0.001	60.58 (6.66)^a^	48.32 (4.56)^c^	52.28 (5.18)^b^	<0.001
BMI (kg/m^2^)	23.85 (2.76)^a^	21.08 (1.72)^b^	23.46 (2.30)^a^	<0.001	24.14 (1.56)^a^	19.15 (1.55)^c^	20.75 (1.69)^b^	<0.001

**Table:** (b) Sample B

Sample B (n = 716)	Male (n = 252)				Female (n = 464)			
	TE (n = 101)	SE (n = 79)	SY (n = 72)	*P* value	TE (n = 145)	SE (n = 156)	SY (n = 163)	*P* value
AGE (y)	31.63 (6.12)	30.85 (5.89)	30.94 (6.27)	0.670	30.83 (6.23)	31.86 (5.77)	31.53 (5.81)	0.326
Height (cm)	174.26 (5.72)	172.70 (5.92)	173.28 (5.30)	0.212	161.41 (4.98)	161.03 (5.07)	160.41 (5.44)	0.239
Weight (kg)	79.37 (10.73)^a^	66.52 (9.76)^b^	69.66 (8.22)^b^	<0.001	63.00 (9.42)^a^	51.03 (5.21)^c^	54.19 (7.20)^b^	<0.001
BMI (kg/m^2^)	26.09 (2.83)^a^	22.26 (2.70)^b^	23.18 (2.36)^b^	<0.001	24.20 (3.59)^a^	19.67 (1.74)^c^	21.05 (2.43)^b^	<0.001

Sample A: native Japanese, Sample B: native Koreans; TE: Tae-Eum, SE: So-Eum, SY: So-Yang.

Data were presented as the mean (standard deviation).

Sample A:* P* values were calculated using Kruskal-Wallis test. Post hoc comparisons using Mann–Whitney.

Sample B: *P* values were calculated using one-way ANOVA. Post hoc comparisons using Bonferroni or Dunnett T3.

a, b, c: Significant difference between the groups, in which the value descends by a and b followed by c.

**Table 4:** Male facial feature variables with differences among the samples and Sasang types.

	Samples			Sasang types				Sample *∗*
								Sasang types
Facial variables	Sample A	Sample B	*P* value	TE	SE	SY	*P* value	*P* value
FA_18_17_43	77.55 (1.16)	83.12 (0.50)	<0.001	82.74 (0.98)^a^	80.68 (0.77)^ab^	77.58 (1.29)^b^	0.006	0.160
FA_118_117_143	78.39 (1.08)	82.81 (0.47)	<0.001	82.96 (0.92)^a^	80.48 (0.72)^ab^	78.37 (1.21)^b^	0.008	0.079
FArea02_aD	6787.01 (114.31)	7266.28 (49.14)	<0.001	7245.54 (96.90)^a^	7064.10 (76.28)^ab^	6770.29 (127.62)^b^	0.012	0.074
FArea03_aD	3831.44 (72.95)	4089.65 (31.36)	0.002	4119.47 (61.84)^a^	3948.55 (48.68)^ab^	3813.62 (81.45)^b^	0.008	0.288
FDH_25_125	97.86 (0.92)	104.46 (0.39)	<0.001	102.63 (0.78)^a^	101.65 (0.61)^ab^	99.20 (1.02)^b^	0.027	0.123
FA_18_17_25	128.72 (1.21)	133.30 (0.52)	0.001	133.14 (1.02)^a^	131.89 (0.81)^a^	127.99 (1.35)^b^	0.009	0.162
FA_118_117_125	127.94 (1.16)	132.30 (0.50)	0.001	132.27 (0.98)^a^	130.84 (0.77)^ab^	127.26 (1.29)^b^	0.008	0.132
FAis_18_17	32.03 (0.80)	29.47 (0.34)	0.005	28.89 (0.68)^b^	30.52 (0.53)^ab^	32.85 (0.89)^a^	0.002	0.265
FAi_118_117	31.71 (0.82)	29.48 (0.35)	0.015	29.01 (0.69)^b^	30.42 (0.55)^ab^	32.35 (0.91)^a^	0.014	0.230
PD_7_77	3.15 (0.28)	2.36 (0.12)	0.011	2.60 (0.24)^ab^	2.39 (0.18)^b^	3.28 (0.31)^a^	0.048	0.224
PDH_12_14	19.48 (0.40)	20.66 (0.17)	0.009	20.58 (0.34)^a^	20.53 (0.27)^a^	19.11 (0.45)^b^	0.015	0.099

Sample A: native Japanese, Sample B: native Koreans; TE: Tae-Eum, SE: So-Eum, SY: So-Yang.

Data were presented as adjusted mean (standard error)

*P* values were calculated using one-way ANCOVA. Post hoc comparisons using Bonferroni.

a, b, c: Significant difference between the groups, in which the value descends by a and b followed by c.

ab: no significant difference among the groups.

**Table 5:** Female facial feature variables with differences among the samples and Sasang types.

	Samples			Sasang types				Sample *∗*
								Sasang types
Facial variables	Sample A	Sample B	*P* value	TE	SE	SY	*P* value	*P* value
FDV_52_50	75.28 (0.65)	71.83 (0.20)	<0.001	73.82 (0.74)^ab^	74.36 (0.53)^a^	72.49 (0.39)^b^	0.011	0.105
FDV_81_50	28.45 (0.38)	26.07 (0.12)	<0.001	27.76 (0.43)	27.42 (0.30)	26.62 (0.22)	0.018	0.103
FA_18_17_43	77.98 (0.96)	75.52 (0.30)	0.016	78.53 (1.08)^a^	75.10 (0.77)^b^	76.63 (0.57)^ab^	0.029	0.816
FArea03_aD	3788.77 (64.90)	3525.75 (20.10)	<0.001	3794.18 (73.24)^a^	3631.84 (52.16)^ab^	3545.76 (38.50)^b^	0.009	0.073
FD_17_26	8.89 (0.18)	10.02 (0.06)	<0.001	9.03 (0.21)^b^	9.80 (0.15)^a^	9.53 (0.11)^ab^	0.010	0.090
FAis_18_17	31.57 (0.69)	35.51 (0.22)	<0.001	32.41 (0.78)^b^	34.70 (0.56)^a^	33.52 (0.41)^ab^	0.044	0.620

Sample A: native Japanese, Sample B: native Koreans; TE: Tae-Eum, SE: So-Eum, SY: So-Yang.

Data were presented as adjusted mean (standard error)

*P* values were calculated using one-way ANCOVA. Post hoc comparisons using Bonferroni.

a, b, c: Significant difference between the groups, in which the value descends by a and b followed by c.

ab: no significant difference among the groups.

## Data Availability

The data used to support the findings of this study are available from the corresponding author upon request.
